# Influence of acclimatization time on parameters of barometric whole-body plethysmography in healthy adult cats

**DOI:** 10.1371/journal.pone.0299252

**Published:** 2024-03-12

**Authors:** Petra Benz, Yury Zablotski, Bianka Schulz

**Affiliations:** Clinic of Small Animal Medicine, Ludwig Maximilian University of Munich, Munich, Germany; University of Wisconsin-La Crosse, UNITED STATES

## Abstract

**Background:**

Pulmonary function testing by barometric whole-body plethysmography (BWBP) is a long-established and well-accepted, non-invasive investigative procedure in cats.

**Hypothesis/Objectives:**

To evaluate, if different acclimatization times influence the measurement parameters of BWBP in healthy adult cats.

**Animals:**

48 healthy adult cats.

**Methods:**

In the prospective observational study, healthy cats were placed in a measuring chamber and BWBP was performed over 30 minutes. Parameters obtained during the three measurement units of 10 minutes each (T1, T2 and T3) were compared.

**Results:**

All measurement parameters except for tidal volume per body weight changed significantly (p<0.05) over the three time periods. From T1-T2, the parameters minute volume per body weight (p<0.001), peak inspiratory flow per body weight (p<0.001), peak expiratory flow per body weight (p = 0.002), pause (p = 0.03), enhanced pause (p = 0.03) and quotient of peak expiratory flow divided by expiratory flow at end expiratory volume plus 50% tidal volume (p = 0.03) changed significantly. From the time interval T2-T3, only respiratory rate (p = 0.02), inspiratory time (p = 0.02), expiratory time (p = 0.04), and relaxation time (p = 0.01) changed significantly. All measurement parameters except for tidal volume per body weight changed significantly (p<0.05) between T1 and T3. Age had a significant influence on all parameters except for peak expiratory flow per body weight and peak inspiratory flow per body weight. The parameters were not influenced by sex.

**Conclusion and clinical importance:**

All measurement parameters except tidal volume per body weight were significantly affected by acclimatization time. Controlling for age and sex, there was still a significant influence of acclimatization time on all parameters except for tidal volume per body weight. Standardization of the acclimatization time for future studies would be appropriate in order to maintain comparability.

## Introduction

Barometric whole-body plethysmography (BWBP), a diagnostic tool for non-invasive lung function measurement, has been the subject of many studies in laboratory animals [1–3], cats [4–6], dogs [7, 8], and pigs [9] in recent years. It is mainly used to diagnose obstructive airway disease, to assess airway reactivity, and to monitor the response to therapy [10]. Originally developed for human infants [11], it is also particularly well tolerated by cats [12], since it can be performed without manipulation in awake animals. For this purpose, the cats are placed in an airtight, transparent plexiglas chamber. Due to pressure differences in the chamber during the breathing cycle, respiratory parameters can be measured and recorded. The pressure changes are generated by two phenomena. First, thoracic motion causes gas expansion and compression [13]. In addition, the inhaled air is humidified and warmed during inspiration, leading to expansion of the air [11, 14]. During expiration, the air is cooled and condensed, leading to contraction [11, 14].

However, since it is not possible for animals to breathe actively through a mouthpiece as it is performed in humans, the plethysmographic measurement must be adapted in animals [10, 12]. The measurement takes place indirectly via the chamber the patient is placed in, which is why the measured signals are referred to as “pseudo-flows” [15].

In various studies on lung function testing, both in human and in companion animals, it has been shown that there are various factors that influence the measurement parameters of BWBP [16, 17]. Previously known factors include somatic growth [18–20], sex [16, 18, 20], age [4, 21], circadian rhythm [18], body mass index [17, 22, 23] and brachycephaly [24, 25].

In contrast to human medicine, the situation in the plethysmographic chamber cannot be explained to animals and they have to get habituated to it before measurement. In cats, the influence of this acclimatization period on BWBP parameters hasn’t been studied yet. In laboratory animals, however, it has already been shown that insufficient acclimatization times can lead to variability in the measurement parameters [3]. Thus, it is recommended that mice first become accustomed to the situation before the actual measurement begins [3]. In studies using animal models, the animals were first placed in the chamber several times for a few minutes before the actual measurements were committed [18]. However, this is usually not possible in clinical trials in companion animals. In different studies in cats the acclimatization time varied from 1–20 minutes [6, 22, 26, 27] or no information was given at all [4, 5, 28]. In addition, it makes a difference for respiratory parameters whether cats are evaluated at home or in a hospital setting [29, 30]. Thus, there could be significant variations in the parameters between the different clinical studies and the ones performed in animal models.

We hypothesized that different acclimatization times have significant influence on the measurement parameters of BWBP in clinically healthy cats and that therefore standard protocols for clinical studies using BWBP in healthy cats are necessary.

## Material and methods

### Ethical approval

All procedures were approved by the Ethics Committee of the Centre for Clinical Veterinary Medicine of LMU Universitiy of Munich (211-07-04-2020). Owner consent was given for all participants.

### Study population

All 48 cats were included in the prospective observational study between May 2020 and September 2021. The participants belonged to co-workers and students or were presented to the clinic for annual health screening. Before each BWBP-measurement, the cats were examined clinically and their health status was determined. All cats were clinically healthy and didn’t have a history of respiratory signs. Inclusion criterion was a minimum age of one year, as somatic growth in cats is known to influence the measurement parameters of the BWBP [18]. Since previous studies showed that obesity has an influence on BWBP parameters too [22], only cats with a normal body condition score (range 3/9 to 6/9) were included.

### Study design

The clinical examination was performed before plethysmographic measurement. Subsequently, lung function parameters were measured over 30 minutes in the chamber of the plethysmograph. All measurements took place in a quiet separate room and the investigator was present at all times.

### Barometric whole-body plethysmography

Lung function was assessed by non-invasive BWBP (Buxco FinePointe Small Animal Whole Body Plethysmograph, Data Science International DSI, New Brighton, Minnesota, USA).

The permanent ventilation of the chamber was ensured by means of bias flow (Buxco® Multi-function Bias Flow, Data Science International (DSI), New Brighton, Minnesota, USA). Sieve pneumotachographs were connected to the chamber, which were responsible for the flow of air in and out of the chamber, creating air resistance. A pressure transducer (Halcyon™ pneumotach, Data Science International (DSI), New Brighton, Minnesota, USA) was also connected, which recorded pressure changes in the chamber (called “box flow”).

The chamber signal measured by the pressure transducer was amplified and digitised by a preamplifier (Buxco® QT Digital Preamplifier, Data Science International (DSI), New Brighton, Minnesota, USA) and forwarded to a computer with an associated software program for analysis (Buxco® FinePointe Small Animal Whole Body Plethysmograph, Data Science International (DSI), New Brighton, Minnesota, USA).

Possible atmospheric noise from outside the room (e.g., dogs barking, slamming doors) was reduced by the pneumotach. Sniffing, vocalization or movement can cause artifacts in waveforms. The FinePointe program automatically removed these artifacts.

Before each measurement, a calibration was performed according to the manufacturer’s recommendation by injecting 50 ml of room air into the chamber. The non-sedated, awake and freely moving cats were then placed into the transparent plexiglass chamber within a transport box, where they spent 30 minutes. Three consecutive measurement periods of 10 minutes each were performed. Parameters measured during the three intervals 0–10 min (T1), 10–20 min (T2) and 20–10 min (T3) were then compared.

All parameters measured by BWBP are listed in Table 1.

**Table 1:** BWBP parameters with units and description.

BWBP parameter	unit	description
**RR**	breaths/min	respiratory rate
**Ti**	s	inspiratory time
**Te**	s	expiratory time
**TV/BW**	ml/kg	tidal volume per body weight
**MV/BW**	ml/min/kg	minute volume per body weight
**PIF/BW**	ml/s/kg	peak inspiratory flow per body weight
**PEF/BW**	ml/s/kg	peak expiratory flow per body weight
**Tr**	ms	relaxation time; time at which 65% of tidal volume is exhaled
**PAU**	unitless	pause, [(Te/Tr)- 1]
**Penh**	unitless	enhanced pause, [((Te/Tr) - 1)x(PEF/PIF)]
**PEF/EF50**	unitless	quotient of peak expiratory pseudo-flow and expiratory flow at end expiratory volume plus 50% tidal volume

min: minute, s: second, ml: millilitre, kg: kilogram, ms: millisecond

### Statistical analysis

All BWBP measurement results were automatically transferred to Microsoft Excel.

The Shapiro-Wilk-test was performed to verify the normal distribution. All data were not normally distributed. Due to the presence of repeated measures the Friedman-test was used to compare the parameters of all cats between the three time periods T1, T2 and T3.

Each of the BWBP parameters was modeled for the interaction between time and age and time and sex using a generalized linear mixed effects model (GLMM). GLMM with individual animal as a random effect was chosen for analysis due to the presence of repeated measures. The following model assumptions were always checked: (1) the normality of residuals was checked by the Shapiro–Wilk normality test, (2) the homogeneity of variances between groups was checked with Bartlett test, and (3) the heteroscedasticity (constancy of error variance) was checked with Breusch–Pagan test. In case assumptions were satisfied, generalized linear mixed effects models were used (R package—lmer). In case assumptions were violated, robust linear mixed effects models were applied (R package—robustlmm). Additionally, both linear and robust linear models were compared amongst each other using six main performance quality indicators: Akaike’s Information Criterion (AIC), Bayesian Information Criterion (BIC), Conditional coefficient of determination R2, Marginal coefficient of determination R2, the intraclass-correlation coefficient (ICC) and Root Mean Square Error (RMSE). The model showing the best combination of predictive (AIC and BIC) and fitting (explanatory, $R^2$, ICC, RMSE) power was preferred. All contrasts between particular groups were assessed after model-fitting by the estimated marginal means using the R package ‘emmeans’ with Tukey p-value correction for multiple comparisons. Results with a P-value less than 0.05 were considered statistically significant. Data analysis was performed using R version 4.2.1 (2022-06-23).

## Results

### Study population

A total of 48 clinically healthy cats were included in the study, consisting of 26 males (24 castrated) and 22 females (20 spayed). The median age was 4.39 ± 4.38 years (range 1 to 16 years). The breeds included European Shorthair (22), Norwegian Forest Cat (6), Siberian Forest Cat (2), Maine Coon (2), British Longhair (1), British Shorthair (3), Birman (4), Bengal (1), British Shorthair mix (1), Norwegian Forest mix (1), Birman x Persian mix (3), Bengal x British Shorthair mix (2). The median BMI was 4/9 (range from 3/9 to 6/9). The cats were divided into three age groups: 1–2 years (A1), 3–7 years (A2) and 8–16 years (A3). Care was taken to ensure an even group size.

The data for all cats are shown in S1 Table.

### Barometric whole-body plethysmography

BWBP was well tolerated by all cats. The mean values of the measurement parameters for each cat and each time period are shown in S2 Table. All measurement parameters, except for TV/BW, were significantly affected by acclimatization time. The measurement results of all cats for all three time periods are shown in Table 2.

**Table 2:** Comparison of BWBP parameters at all three time periods in healthy cats.

parameter	unit	T1	T2	T3	p	p T1-T2	p T2-T3	p T1-T3
**RR**	breaths/min	65.4 (46.6–104.2)	61.3 (40.4–104.9)	56.7 (35.7–95.2)	**<0.001**	0.66	**0.02**	**<0.001**
**Ti**	s	0.4 (0.3–0.5)	0.4 (0.3–0.6)	0.5 (0.3–0.7)	**<0.001**	0.66	**0.02**	**<0.001**
**Te**	s	0.6 (0.3–0.9)	0.6 (0.3–1.0)	0.7 (0.4–1.0)	**<0.001**	0.20	**0.04**	**<0.001**
**TV/BW**	ml/kg	4.2 (3.1–5.4)	4.0 (2.7–5.2)	4.2 (2.8–5.4)	0.26	1.00	0.31	1.00
**MV/BW**	ml/min/kg	257.4 (214.1–325.4)	228.2 (187.5–298.6)	222.8 (174.9–281.9)	**<0.001**	**<0.001**	0.66	**<0.001**
**PIF/BW**	ml/s/kg	16.3 (13.5–19.1)	14.9 (12.4–17.6)	14.7 (11.8–17.0)	**<0.001**	**<0.001**	1.00	**<0.001**
**PEF/BW**	ml/s/kg	9.8 (8.4–12.6)	8.8 (7.2–12.5)	8.7 (7.3–12.8)	**<0.001**	**0.002**	1.00	**<0.001**
**Tr**	ms	0.4 (0.2–0.5)	0.3 (0.2–0.5)	0.4 (0.2–0.6)	**<0.001**	0.92	**0.01**	**<0.001**
**PAU**	unitless	0.77 (0.7–0.9)	0.8 (0.7–0.9)	0.8 (0.7–0.9)	**0.003**	**0.03**	1.00	**0.003**
**Penh**	unitless	0.5 (0.5–0.7)	0.6 (0.5–0.7)	0.6 (0.5–0.7)	**0.003**	**0.03**	1.00	**0.003**
**PEF/EF50**	unitless	1.2 (1.1–1.3)	1.2 (1.1–1.3)	1.2 (1.1–1.3)	**<0.001**	**0.03**	0.07	**<0.001**

Data is reported as median with IQR. Values shown in bold indicate p > 0.05. Fourth last column indicates p value over all three time periods. The corrected values between the different time periods are shown in the last three columns. RR: respiratory rate, Ti: inspiratory time, Te: expiratory time, TV/BW: tidal volume per body weight, MV/BW: minute volume per body weight, PIF/BW: peak inspiratory pseudo-flow per body weight, PEF/BW: peak expiratory pseudo-flow per body weight, RT: relaxation time; time point when 65% of tidal volume is expired, PAU: pause (Te-RT)/RT), Penh: enhanced pause [(PEF/PIF)x((Te/Tr) -1)], PEF/EF50: quotient of peak expiratory pseudo-flow divided by expiratory flow at end expiratory volume plus 50% tidal volume.

From the time period T1-T2, significant changes in the measurement parameters MV/BW, PIF/BW, PEF/BW, Pau, Penh, PEF/EF50 were seen. Between T2 and T3, the parameters RR, Ti, Te, and Tr were significantly different (P<0.05). From the time interval T1 to T3, all measurement parameters except for TV/BW changed significantly.

**Fig 1:**
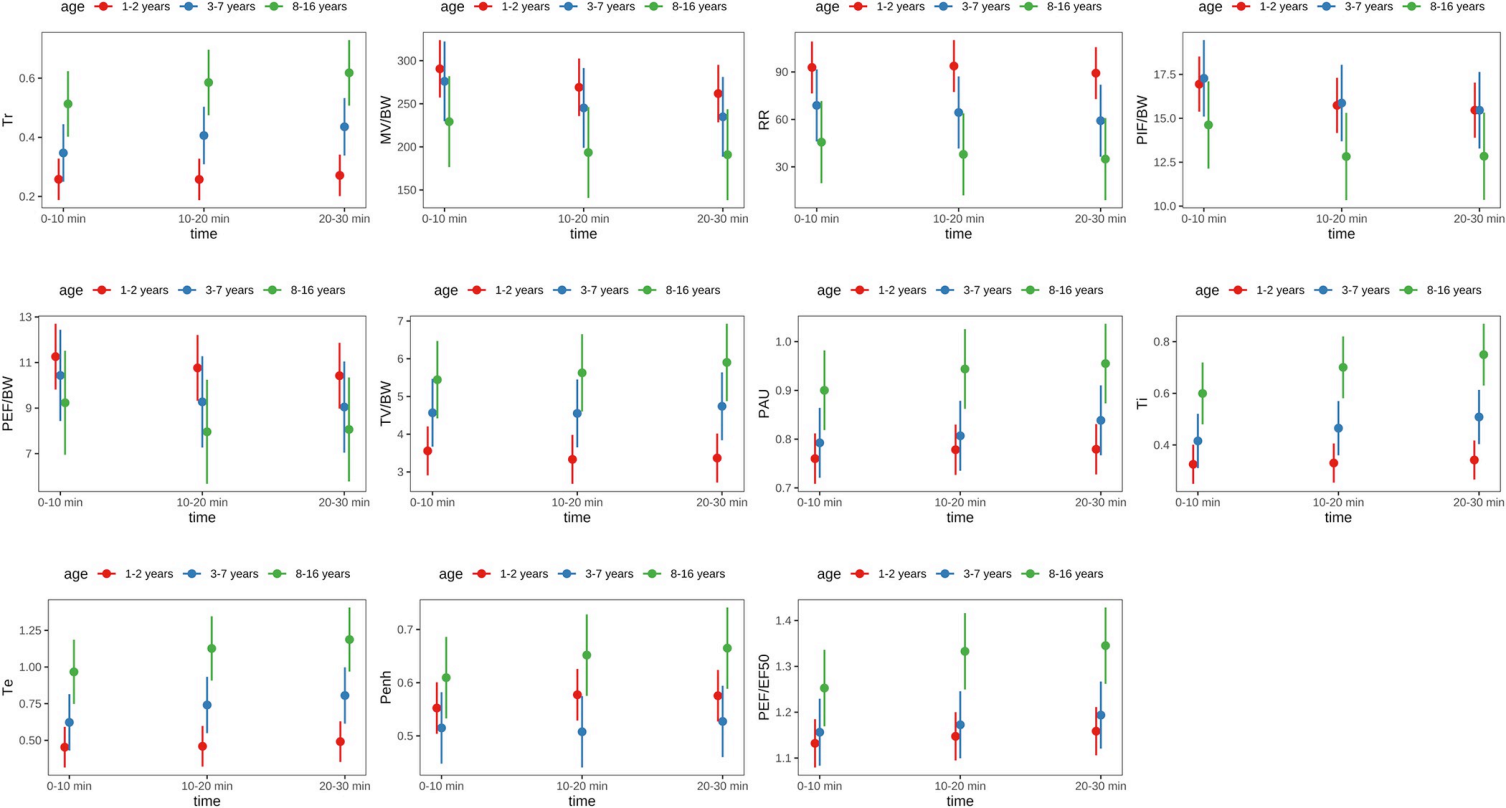
Comparison of the three age groups per time unit for each BWBP parameter.

Using a mixed effects model, it can be shown that age had a significant influence on all BWBP parameters except for PEF/BW and PIF/BW. Details are shown in Table 3 and Fig 1.

**Table 3:** Influence of age subdivided in three age groups at three time points on the parameters of BWBP.

parameter	T1			T2			T3			T1			T2			T3		
	A1	A2	A3	A1	A2	A3	A1	A2	A3	A1-A2 p	A1-A3 p	A2-A3 p	A1-A2 p	A1-A3 p	A2-A3 p	A1-A2 p	A1-A3 p	A2-A3 p
**RR (breaths/min)**	92.8(76.58, 109.1)	68.8 (46.29, 91.4)	45.7 (19.93, 71.4)	93.7 (77.47, 110.0)	64.4 (41.83, 87.0)	37.9 (12.21,63.7)	89.2 (72.96, 105.5)	59.2 (36.63, 81.8)	34.9 (9.22, 60.7)	0.2086	**0.0067**	0.3792	0.0965	**0.001**	0.283	0.0864	**0.0014**	0.3466
**Ti (s)**	0.325 (0.250, 0.400)	0.416 (0.311, 0.520)	0.600 (0.481, 0.718)	0.330 (0.255, 0.405)	0.465 (0.361, 0.569)	0.701 (0.582, 0.819)	0.342 (0.267, 0.417)	0.508 (0.404, 0.612)	0.750 (0.631, 0.869)	0.3523	**<0.001**	0.0581	0.0985	**<0.001**	**0.0096**	**0.0296**	**<0.001**	**0.0077**
**Te (s)**	0.453 (0.16, 0.591)	0.623 (0.432, 0.813)	0.967 (0.750, 1.184)	0.459 (0.322, 0.597)	0.741 (0.551, 0.932)	1.127 (0.909, 1.344)	0.491 (0.354, 0.629)	0.806 (0.615, 0.996)	1.187 (0.970, 1.405)	0.3337	**<0.001**	0.0510	**0.0491**	**<0.001**	**0.0241**	**0.0235**	**<0.001**	**0.0260**
**TV/BW (ml/kg)**	3.56 (2.91, 4.20)	4.57 (3.68, 5.46)	5.44 (4.43, 66.46)	3.33 (2.69, 3.98)	4.55 (3.66, 5.44)	5.63 (4.61, 6.64)	3.37 (2.73, 4.01)	4.74 (3.85, 5.63)	5.90 (4.89, 6.92)	0.1689	**0.0060**	0.4117	0.0759	**<0.001**	0.2645	**0.0384**	**<0.001**	0.2106
**MV/BW (ml/min/kg)**	291 (258, 324)	276 (230 322)	229 (177, 282)	269 (236, 302)	245 (199, 291)	193 (141, 246)	262 (229, 295)	235 (189, 281)	191 (139, 243)	0.8696	0.1269	0.3847	0.6845	**0.0436**	0.3115	0.6178	0.0637	0.4309
**PIF/BW (ml/s/kg)**	16.9 (15.4, 18.5)	17.3 (15.1, 19.4)	14.6 (12.2, 17.1)	15.7 (14.2, 17.3)	15.9 (13.7, 18.0)	12.8 (10.4, 15.3)	15.5 (13.9, 17.0)	15.5 (13.3, 17.6)	12.8 (10.4, 15.3)	0.9677	0.2617	0.2503	0.9947	0.1225	0.1624	1.00	0.1811	0.2594
**PEF/BW (ml/s/kg)**	11.26 (9.83, 12.7)	10.43 (8.45, 12.4)	9.23 (6.97, 11.5)	10.76 (9.33, 12.2)	9.27 (7.29, 11.3)	7.96 (5.70, 10.2)	10.42 (8.99, 11.9)	9.05 (7.06, 11.0)	8.06 (5.80, 10.3)	0.7868	0.2998	0.7144	0.4572	0.100	0.6682	0.5133	0.1951	0.7975
**Tr (ms)**	0.258 (0.189, 0.328)	0.347 (0.251, 0.444)	0.513 (0.403, 0.623)	0.258 (0.188, 0.327)	0.406 (0.310, 0.503)	0.585 (0.475, 0.695)	0.271 (0.202, 0.341)	0.436 (0.339, 0.532)	0.618 (0.508, 0.728)	0.3053	**<0.001**	0.0674	**0.0381**	**<0.001**	**0.0429**	**0.0185**	**<0.001**	**0.0382**
**PAU (unitless)**	0.760 (0.709, 0.811)	0.793 (0.721, 0.864)	0.900 (0.819, 0.981)	0.778 (0.727, 0.830)	0.807 (0.736, 0.878)	0.944 (0.863, 1.025)	0.779 (0.728, 0.830)	0.839 (0.768, 0.910)	0.955 (0.874, 1.036)	0.7482	**0.0116**	0.1224	0.7978	**0.0020**	**0.0339**	0.3796	**<0.001**	0.0862
**Penh (unitless)**	0.552 (0.504, 0.600)	0.515 (0.448, 0.581)	0.609 (0.534, 0.785)	0.577 (0.529, 0.625)	0.508 (0.441, 0.574)	0.652 (0.576, 0,728)	0.575 (0.528, 0.623)	0.527 (0.461, 0.594)	0.665 (0.589, 0.741)	0.6441	0.4253	0.1578	0.2195	0.2343	**0.0142**	0.4805	0.1237	**0.0203**
**PEF/EF50 (unitless)**	1.13 (1.08, 1.18)	1.16 (1.08, 1.23)	1.25 (1.17, 1.34)	1.15 (1.10, 1.20)	1.17 (1.10, 1.25)	1.33 (1.25, 1.42)	1.16 (1.11, 1.21)	1.19 (1.12, 1.27)	1.35 (1.26, 1.43)	0.8561	**0.0416**	0.1987	0.8447	**<0.001**	**0.0119**	0.7213	**<0.001**	**0.0190**

Data for each age group and time period are presented as means with lower confidence limit and upper confidence limit. The confidence interval was set at 95%. The p-values for the comparisons of the measurement results as a dependency of each age group at each time point are shown in the last nine columns. Values in bold indicate p<0.05.

A1 = 1–2 years, A2 = 3–7 years and A3 = 8–16 years.

When examining the influence of sex on the parameters of BWBP, no significant influence could be found (Table 4).

**Table 4:** Influence of sex at three time points on the parameters of BWBP.

Parameter	T1		T2		T3		T1	T2	T3
	female	male	female	male	female	male	female-male p-value	female-male p-value	female-male p-value
**RR (breaths/min)**	74.3 (54.8, 93.7)	75.0 (57.1, 92.9)	69.9 (50.4, 89.4)	74.5 (56.6, 92.4)	63.9 (44.4, 83.4)	71.5 (53.5, 89.4)	0.9569	0.7327	0.5771
**Ti (s)**	0.423 (0.318, 0.529)	0.405 (0.308, 0.502)	0.470 (0.364, 0.575)	0.426 (0.329, 0.523)	0.513 (0.408, 0.619)	0.438 (0.342, 0.535)	0.8002	0.5515	0.3068
**Te (s)**	0.642 (0.449, 0.834)	0.594 (0.417, 0.771)	0.724 (0.532, 0.917)	0.635 (0.458, 0.811)	0.785 (0.592, 0.977)	0.668 (0.491, 0.845)	0.7218	0.5010	0.3790
**TV/BW (ml/kg)**	4.34 (3.54, 5.13)	4.16 (3.43, 4.89)	4.20 (3.40, 4.99)	4.09 (3.36, 4.83)	4.48 (3.69, 3.35)	4.09 (3.35, 4.82)	0.7484	0.8533	0.4731
**MV/BW (ml/min/kg)**	272 (236, 308)	273 (239, 306)	237 (201, 273)	253 (219, 286)	229 (192, 213)	246 (213, 280)	0.9681	0.5396	0.4851
**PIF/BW (ml/s/kg)**	16.6 (14.9, 18.3)	16.5 (14.9, 18.0)	14.8 (13.1, 16.5)	15.4 (13.8, 17.0)	14.5 (12.8, 16.2)	15.2 (13.6, 16.7)	0.9113	0.5939	0.5818
**PEF/BW (ml/s/kg)**	10.43 (8.93, 11.9)	10.59 (9.21, 12.0)	9.33 (7.82, 10.8)	10.00 (8.62, 11.4)	9.09 (7.59, 10.6)	9.79 (8.41, 11.2)	0.8752	0.5182	0.5025
**Tr (ms)**	0.353 (0.257, 0.448)	0.328 (0.241, 0.416)	0.391 (0.296, 0.486)	0.345 (0.257, 0.432)	0.425 (0.330, 0.520)	0.335 (0.268, 0.443)	0.7137	0.4839	0.2936
**PAU (unitless)**	0.797 (0.733, 0.861)	0.805 (0.746, 0.864)	0.825 (0.761, 0.889)	0.821 (0.762, 0.880)	0.834 (0.770, 0.898)	0.833 (0.774, 0.892)	0.8531	0.9312	0.9891
**Penh (unitless)**	0.544 (0.488, 0.599)	0.564 (0.513, 0.615)	0.568 (0.513, 0.624)	0.579 (0.527, 0.630)	0.574 (0.519, 0.630)	0.587 (0.536, 0.638)	0.5944	0.7929	0.7360
**PEF/EF50 (unitless)**	1.17 (1.11, 1,23)	1.16 (1.11, 1.22)	1.20, (1.14, 1.26)	1.18 (1.12, 1.24)	1.22 (1.16, 1.28)	1.19 (1.13, 1.25)	0.8814	0.6313	0.4974

Data are shown as mean with lower confidence limit and upper confidence limit. The confidence interval was set at 95%. The p-values for the comparison of the measurement results as a dependency of gender at the individual time points are shown in the last three columns. Values in bold indicate p<0.05

There was a significant influence of acclimatization time on all BWBP parameters except for TV/BW, even after controlling for age and sex.

## Discussion

The study was able to show that different acclimatization times significantly influenced parameters of BWBP in healthy cats. Except for the parameter TV/BW, all other parameters changed significantly. In previous studies in cats, acclimatization times were not standardized or no information was given about the time cats spent in the measuring chamber before measurement started [4–6, 22, 26–28]. However, it is possible that in studies where the acclimatization time was not specified, it was individually adjusted to the behaviour of each cat to choose time periods for measurement, when animals seemed calm and relaxed.

Particularly notable in the present study was the significant increase in Penh (enhanced pause) over time between T1 and T2 and T1 and T3. Penh is a unitless parameter calculated using the formula [((Te/Tr) - 1) x (PEF/PIF)]. Many authors state that an increased Penh value in cats indicates increased airway resistance [4–6, 15, 31]. It has also been found to be a particularly stable factor that is not influenced by age, gender or weight [18, 22].

However, many authors questioned its function as an indicator of increased airway resistance over the last few years [1, 32–34]. An increase in Penh is induced by a rapid and steep drop in chamber pressure [32]. *In vivo*, this is only to be expected in the case of rapid compression of the lungs through active expiration (e.g., during vocalisation in neonatal mice[35, 36]) [32]. Active expiration causes a decline in temperature in the chamber, resulting in a drop in pressure [32]. Therefore, Huelsmann and co-workers described Penh rather as an indicator for active expiration. In their artificial lung model, they found that a significant pressure drop in the chamber requires not only increased airway resistance, but also active expiration. In turn, they were able to document a rapid drop in pressure during expiration independent of the level of airway resistance [32]. These results show the influence of the timing and magnitude of the pressure drop on Penh. Therefore, this parameter should be interpreted with great caution, if measuring protocols have not been standardized in studies. It is also shown that Penh is directly related to the timing and pattern of breathing rather than airway resistance [34]. It can be used as an indicator of airway resistance, if the gas in the chamber has previously been warmed to body temperature and the humidity is preconditioned [34]. Because in the present study, humidity in the chamber was not standardized and temperature was relatively stable, but the chamber was not preheated to body temperature, these factors might have influenced the measurements considering a slow temperature rise in the box to be expected due to the body temperature of the animal. In addition, variations of the measuring times during the day could have had an influence on the parameter, because Penh is known to be affected by the circadian rhythm [18]. To the best of the authors’ knowledge, no reference values for Penh in healthy cats have been reported to date, but the values obtained in this study during all three time periods are lower than those measured in cats with respiratory disease [6, 12, 23]. Compared to data obtained by our group in a previous study investigating cats with feline lower airway disease using the same plethysmograph, program and settings [37], Penh was higher in diseased cats compared to the healthy ones in present study. However, on re-check examinations after initiation of anti-inflammatory therapy, Penh values of cats with airway disease in the previous study were within the range determined for healthy cats in the present study [37].

In the present study there was a significant decrease in respiratory rate (RR) over the three time periods. At baseline, RR was at a median of 65 breaths/minute, consistent with values from a study in which healthy cats had a median RR of 64 breaths/minute in a clinical setting [30], which decreased to a median of 57 in the third measuring period. Values of all three measuring phases are considerably higher compared to the physiological RR defined for healthy cats with less than 30 breaths/minute [38]. This elevation can be explained by the increased level of stress caused by travel and the unfamiliar environment [29, 30]. As patients became accustomed to the plethysmographic chamber and felt more familiar with the situation over time, the RR decreased. Inspiratory time Ti and expiratory time Te are parameters directly related to the RR, since the respiratory cycle is composed of both components. With a decrease of the RR over the three time periods, Te and Ti were seen to become longer. It is well known that Te and Ti are also influenced by stress [39, 40]. In a study investigating breathing patterns in stressful situations in humans, Te and Ti were shown to decrease significantly [39]. In contrast in the present study, the expected reduction of stress over time led to a prolongation of both parameters.

The parameter pause (PAU) is calculated with the formula PAU = (Te/Tr)-1 and thus dependent on Te and Tr. As both parameters change, there is also a significant increase in PAU over time.

Consistent with data from a study investigating the influence of the acclimatization time on BWBP-parameters in healthy conscious mice [3], a significant change in peak inspiratory flow (PIF) and peak expiratory flow (PEF) was detected in the present study over time. PEF and PIF stabilised in mice after approximately 10 minutes. In the present study in cats, the values were still changing after 20 minutes. Both parameters belong to the so-called pseudo-tidal breathing flow-volume loop parameters (pTBFVL) and are usually expressed as PEF/BW and PIF/BW because of the known influence of the body weight [12]. These flow signals were originally measured in cats and dogs using a face mask and recorded via a pneumotachograph with pressure transducer [41]. As this method is very difficult to use in cats, measurements were modified to be taken with the plethysmograph. In the plethysmographic chamber, flow signals are created by nasal airflow and thoracic movements and are therefore slightly different from those obtained with a face mask and pneumotachograph [12, 41, 42], which is why they are referred to as pseudo-flow signals [15]. Meowing, purring or movement can distort the flow measurement and create artefacts in the loops. Therefore, only loops without artefacts were used in the study that applied the method with the face mask in cats [41]. In the present study, disturbances such as movement and vocalisation were automatically removed from the data by the FinePointe programme. However, a study also using BWBP in cats revealed that artefacts can still occur despite automatic programmes [26]. Body movements of the participants caused significant deviations, especially in the parameters PEF and PIF [26]. Especially at the beginning of the measurements in the present study, many cats were more restless and moved around more frequently, which could have led to artefacts in the flow signals and might have caused significant changes of the pTBFVL over the three time periods.

A significant decrease of minute ventilation per body weight (MV/BW) could be observed in the present study over time. As a consequence of the influence of stress and anxiety on the respiratory cycle, an increase in minute ventilation can be observed, as described in healthy humans [40] and in mice before [3]. Based on data of the present study, an adequate acclimatization time can be recommended to reduce stress in cats as well, leading to a decrease in MV/BW as a consequence.

Tidal volume (TV) is also the only parameter in the current study that was not influenced by acclimatization time. It is defined as the volume of air per breath, usually being around 10–20 ml/kg in cats [6]. Previous studies have already identified TV as a very stable parameter in cats [43] and in laboratory animals [3]. However, TV was the only parameter in the present study that remained stable over all three measuring periods.

Using a mixed effects model, a significant influence of age on all parameters except PEF/BW and PIF/BW was found. Since it is known from several mammalian studies that airway reactivity changes with age [4, 44–48], these results are not surprising. The significant influences of age on BWBP parameters is consistent with results from a previous study in which RR increased and TV/BW decreased in young compared to old cats [4]. Penh, a parameter frequently used to assess bronchoconstriction [2, 31, 49], was also significantly influenced by age in the present study. Older cats aged 8–16 years had a significantly higher Penh compared to those aged 3–7 years. In contrast, Hirt and co-workers could not find an influence of age on Penh. However, the age group of 3 to 11-year-old cats that were evaluated in his study, was not represented in the present study. Comparing the Penh values of the old cats in the current study with the values of the old cats in the former study, reveals similar results (0.643 ± 0.050) [4]. Compared to studies of basal Penh values in cats with bronchial disease, the mean values in the present study were only slightly lower and in a similar range [31, 37]. A correlation between age and airway obstruction has been suggested in human studies [46, 50]. However, a veterinary study showed a significant decrease in airway resistance (PCPENH300) with increasing age after provocation [4]. Since a clinical influence of the factor age on Penh cannot be excluded, the authors recommend, based on the results of the present study, that age should be taken into account when using BWBP to assess respiratory function in cats.

No significant influence of sex on BWBP parameters was found in the present study. These findings are in agreement with findings of other studies investigating the influence of sex on BWBP parameters in healthy cats [4, 18]. A significant sex-dependent influence was only found for the weight-specific volume and pseudoflow parameters in a previous study, as male cats have a larger lung volume due to their higher weight [18]. In the present study, these parameters (TV, MV, PEF and PIF) were already calculated by taking weight into account.

The study had several limitations. The circadian rhythm can have an influence on several parameters, including Penh, RR, Ti, Te, Tr, PIF and PEF [18]. The measurements in the current study were carried out at different times of the day and therefore could have been affected by this condition. In addition, most parameters are influenced by humidity and temperature and their fluctuations in the plethysmographic chamber [9, 18, 34, 42]. In the present study, neither the temperature nor the humidity could have been kept constant. For future studies, a presetting taking these influencing factors into account would be advisable.

## Conclusion

The study indicates that most parameters measured by BWBP in healthy adult cats are strongly influenced by acclimatization time. Taking age and sex into account, there is still a significant influence of acclimatization time on all BWBP parameters except for TV/BW. Standardization of the measurement protocols for further studies should therefore be considered to ensure comparability of the results. Since many parameters still changed significantly between the T1 and T3 time periods, an acclimatization time of 20 minutes can be recommended. Alternatively, the well-being and stress level of the cats in the plethysmographic chamber could possibly be assessed using an ethogram, and the acclimatization time could be adjusted individually based on the temperament and well-being of each cat.

Since age-related significant differences were found for all BWBP parameters except PIF/BW and PEF/BW, the age of the cats should be taken into account when evaluating pulmonary function diagnostics in future studies.

## Supporting information

S1 Table Data of all 48 cats. (PDF)

S2 Table Mean values of each BWBP measurement parameter for each cat and each time period. (PDF)
