# Supplementary material for: Influence of acclimatization time on parameters of barometric whole-body plethysmography in healthy adult cats
Source: PLoS One. 2024 Mar 12;19(3):e0299252. doi: 10.1371/journal.pone.0299252 (PMC10931434; doi:10.1371/journal.pone.0299252)
Supplement: S1 Table — (PDF) [file pone.0299252.s001.pdf]

S1 Table. Data of all 48 cats included in the study.

CAT NR.	AGE (YEAR)	SEX	BREED	WEIGHT (KG)	BODY CONDITION SCORE
1	**1**	**male castrated**	**Norwegian Forest Cat**	**6.6**	**5/9**
2	**1**	**male castrated**	**Norwegian Forest Cat**	**3.97**	**4/9**
3	**1**	**female spayed**	**Norwegian Forest Cat**	**6.2**	**5/9**
4	**1**	**male castrated**	**Norwegian Forest Cat**	**6.2**	**5/9**
5	**1**	**male castrated**	**European Shorthair**	**4.5**	**4/9**
6	**1**	**female spayed**	**European Shorthair**	**3.1**	**3/9**
7	**1**	**female spayed**	**European Shorthair**	**4.6**	**4/9**
8	**1**	**male castrated**	**European Shorthair**	**5.35**	**5/9**
9	**1**	**male castrated**	**European Shorthair**	**4.25**	**5/9**
10	**1**	**male castrated**	**European Shorthair**	**4.35**	**4/9**
11	**1**	**male castrated**	**European Shorthair**	**4.5**	**4/9**
12	**1**	**male intact**	**Bengal x British Shorthair mix**	**5.0**	**5/9**
13	**1**	**male intact**	**Bengal x British Shorthair mix**	**5.0**	**4/9**
14	**9**	**female spayed**	**Siberian Forest Cat**	**4.57**	**4/9**

15	**7**	**female spayed**	**Siberian Forest Cat**	**4.2**	**4/9**
16	**1**	**male castrated**	**Maine Coon**	**4.4**	**5/9**
17	**1**	**female spayed**	**British Longhair**	**3.3**	**4/9**
18	**1**	**male castrated**	**European Shorthair**	**3.85**	**4/9**
19	**1**	**male castrated**	**British Shorthair**	**3.8**	**5/9**
20	**1**	**male castrated**	**European Shorthair**	**4.9**	**5/9**
21	**1**	**female spayed**	**European Shorthair**	**3.45**	**4/9**
22	**1**	**female intact**	**British Shorthair**	**3.4**	**4/9**
23	**1**	**male castrated**	**British Shorthair**	**4.4**	**5/9**
24	**1**	**male castrated**	**European Shorthair**	**4.2**	**4/9**
25	**16**	**male castrated**	**European Shorthair**	**5.5**	**5/9**
26	**7**	**female spayed**	**European Shorthair**	**5.4**	**5/9**
27	**3**	**male castrated**	**European Shorthair**	**5.3**	**5/9**
28	**7**	**male castrated**	**European Shorthair**	**4.8**	**5/9**
29	**8**	**female spayed**	**European Shorthair**	**3.9**	**4/9**
30	**1**	**female intact**	**Norwegian Forest Cat**	**5.1**	**5/9**

31	**1**	**female intact**	**Norwegian Forest Cat**	**5.1**	**5/9**
32	**15**	**female spayed**	**Maine Coon**	**5.3**	**3,5/9**
33	**15**	**female spayed**	**Birman**	**3.5**	**3,5/9**
34	**13**	**female spayed**	**British Shorthair mix**	**6.2**	**6/9**
35	**9**	**female spayed**	**European Shorthair**	**4.5**	**5/9**
36	**4**	**male castrated**	**Norwegian Forest Cat mix**	**5.4**	**5/9**
37	**11**	**female spayed**	**European Shorthair**	**3.7**	**4/9**
38	**3**	**female spayed**	**Birman**	**3.7**	**3/9**
39	**3**	**male castrated**	**Birman**	**4.6**	**4/9**
40	**4**	**male castrated**	**Birman x Persian mix**	**4.9**	**4/9**
41	**5**	**male castrated**	**European Shorthair**	**3.7**	**3,5/9**
42	**8**	**male castrated**	**European Shorthair**	**4.9**	**5/9**
43	**8**	**female spayed**	**European Shorthair**	**3.6**	**4/9**
44	**4**	**female intact**	**Birman x Persian mix**	**3.2**	**4/9**
45	**7**	**female spayed**	**Birman**	**3.1**	**4/9**
46	**6**	**female spayed**	**European Shorthair**	**3.7**	**4/9**

47	**2**	**male castrated**	**Bengal**	**5.1**	**4/9**
48	**4**	**male castrated**	**Birman x Persian mix**	**5.2**	**4/9**
