# Supplementary material for: Influence of acclimatization time on parameters of barometric whole-body plethysmography in healthy adult cats
Source: PLoS One. 2024 Mar 12;19(3):e0299252. doi: 10.1371/journal.pone.0299252 (PMC10931434; doi:10.1371/journal.pone.0299252)
Supplement: S2 Table — (PDF) [file pone.0299252.s002.pdf]

S2 Table. Mean values of all BWBP parameters for each cat and each time period.

CAT NR.	TIME	RR	PENH	PAU	TI	TE	TR	TV/BW	MV/BW	PIF/BW	PEF/BW	PEF/EF50
**1**	T1	131.73	0.50	0.72	0.23	0.30	0.18	1.95	214.50	12.92	8.36	1.08
**1**	T2	103.47	0.47	0.73	0.26	0.36	0.21	2.10	196.42	12.00	7.48	1.09
**1**	T3	86.40	0.46	0.71	0.30	0.42	0.25	2.01	164.18	10.06	6.25	1.08
**2**	T1	21.55	0.82	1.41	0.95	2.06	0.88	9.90	211.27	16.32	8.09	1.16
**2**	T2	22.11	0.87	1.38	0.97	1.86	0.82	10.14	223.39	16.44	9.00	1.17
**2**	T3	21.27	0.99	1.63	0.98	2.12	0.83	10.00	206.33	16.35	8.31	1.21
**3**	T1	149.35	0.44	0.64	0.19	0.23	0.14	1.78	249.81	14.53	9.72	1.03
**3**	T2	142.07	0.44	0.65	0.20	0.25	0.15	1.61	212.38	12.31	8.28	1.04
**3**	T3	128.09	0.43	0.65	0.21	0.27	0.17	1.56	193.62	11.25	7.32	1.03
**4**	T1	59.26	0.45	0.84	0.49	0.70	0.38	3.54	189.67	14.04	6.83	1.24
**4**	T2	86.54	0.47	0.79	0.29	0.44	0.24	2.45	202.01	12.88	7.37	1.12
**4**	T3	102.93	0.50	0.77	0.25	0.35	0.20	2.16	211.34	12.83	8.09	1.10
**5**	T1	207.87	0.53	0.68	0.14	0.15	0.09	1.58	323.26	18.00	13.85	1.01
**5**	T2	203.53	0.53	0.68	0.14	0.16	0.09	1.51	303.36	17.01	13.00	1.01
**5**	T3	196.58	0.53	0.68	0.15	0.17	0.10	1.65	298.41	16.68	12.77	1.02
**6**	T1	71.00	0.50	0.76	0.36	0.52	0.30	3.73	255.76	15.17	9.30	1.18
**6**	T2	75.10	0.54	0.77	0.34	0.49	0.28	3.56	260.41	15.04	9.78	1.20
**6**	T3	78.58	0.52	0.76	0.34	0.48	0.27	3.85	281.45	16.44	10.48	1.19
**7**	T1	141.06	0.42	0.65	0.20	0.27	0.16	2.32	300.00	17.48	10.85	1.05
**7**	T2	140.17	0.40	0.64	0.19	0.26	0.16	2.04	269.97	15.89	9.73	1.05
**7**	T3	95.85	0.46	0.70	0.30	0.41	0.24	3.48	282.02	16.48	10.25	1.11
**8**	T1	196.88	0.56	0.67	0.16	0.17	0.10	2.12	380.46	20.48	16.56	1.02

**8**	T2	199.38	0.58	0.69	0.15	0.17	0.10	2.05	367.86	19.68	16.31	1.02
**8**	T3	207.35	0.57	0.68	0.15	0.16	0.10	1.97	359.43	19.06	15.68	1.02
**9**	T1	109.36	0.36	0.65	0.23	0.33	0.20	3.23	346.12	21.68	11.88	1.07
**9**	T2	102.62	0.38	0.67	0.25	0.36	0.22	3.66	365.64	22.36	12.35	1.07
**9**	T3	125.35	0.45	0.68	0.21	0.28	0.17	2.95	359.25	20.94	13.36	1.07
**10**	T1	54.29	0.63	0.86	0.46	0.66	0.36	5.01	269.05	15.96	10.26	1.24
**10**	T2	51.93	0.67	0.90	0.48	0.69	0.37	5.11	261.86	15.77	10.40	1.27
**10**	T3	55.00	0.57	0.83	0.45	0.66	0.36	4.92	263.95	16.61	10.07	1.24
**11**	T1	130.89	0.53	0.75	0.22	0.26	0.15	3.44	419.85	24.84	16.48	1.10
**11**	T2	153.92	0.59	0.79	0.19	0.22	0.12	2.36	339.15	19.28	13.86	1.09
**11**	T3	153.64	0.52	0.75	0.18	0.22	0.13	2.33	354.69	21.15	13.99	1.10
**12**	T1	171.28	0.59	0.78	0.17	0.20	0.11	2.61	422.65	23.77	16.90	1.07
**12**	T2	190.76	0.60	0.78	0.15	0.18	0.10	2.18	388.90	21.93	16.42	1.05
**12**	T3	186.39	0.66	0.78	0.16	0.19	0.10	2.48	402.95	22.26	17.75	1.07
**13**	T1	64.86	0.71	0.98	0.39	0.68	0.38	4.15	236.63	16.72	9.18	1.25
**13**	T2	82.43	0.60	0.84	0.33	0.49	0.27	2.98	217.59	13.40	8.45	1.19
**13**	T3	67.66	0.46	0.82	0.33	0.59	0.33	2.58	170.20	11.70	6.07	1.29
**14**	T1	52.79	0.56	0.77	0.52	0.73	0.42	6.06	303.95	18.02	12.10	1.15
**14**	T2	45.74	0.64	0.93	0.56	0.85	0.44	4.80	216.31	13.75	8.69	1.19
**14**	T3	44.50	0.62	0.95	0.58	0.94	0.49	4.85	207.28	13.76	8.40	1.24
**15**	T1	58.46	0.56	0.86	0.47	0.70	0.38	5.48	289.09	19.40	11.58	1.18
**15**	T2	43.13	0.48	0.95	0.53	1.00	0.52	4.39	184.52	14.74	6.49	1.24
**15**	T3	42.23	0.46	0.95	0.55	1.02	0.53	4.46	178.45	14.90	6.39	1.27
**16**	T1	105.92	0.70	0.84	0.26	0.32	0.17	4.47	468.51	25.87	20.52	1.18
**16**	T2	105.52	0.74	0.88	0.25	0.32	0.17	4.20	438.85	24.63	19.82	1.24

**16**	T3	93.25	0.73	0.88	0.28	0.37	0.20	4.32	398.90	22.61	17.67	1.23
**17**	T1	257.46	0.68	0.74	0.13	0.12	0.07	2.85	613.61	32.47	29.27	1.04
**17**	T2	268.77	0.78	0.81	0.12	0.12	0.06	2.51	573.00	30.58	29.63	1.01
**17**	T3	259.17	0.72	0.76	0.12	0.13	0.07	2.68	527.71	28.72	26.30	1.05
**18**	T1	54.52	0.78	0.92	0.50	0.71	0.37	3.62	184.76	11.13	8.26	1.54
**18**	T2	44.53	0.82	1.01	0.59	0.86	0.43	3.70	154.59	10.15	7.13	1.60
**18**	T3	43.50	0.90	1.05	0.65	0.92	0.45	3.88	150.93	10.08	7.54	1.61
**19**	T1	68.23	0.49	0.81	0.36	0.54	0.30	3.89	261.38	17.40	9.78	1.26
**19**	T2	63.79	0.50	0.83	0.38	0.59	0.32	3.93	240.64	16.77	9.26	1.31
**19**	T3	65.84	0.52	0.84	0.38	0.58	0.32	3.98	250.72	17.09	9.68	1.28
**20**	T1	29.97	1.09	1.30	0.82	1.30	0.57	6.63	192.85	12.29	8.43	1.26
**20**	T2	25.43	1.04	1.33	0.89	1.54	0.67	5.81	145.66	10.17	6.38	1.37
**20**	T3	27.91	1.05	1.31	0.88	1.46	0.64	6.55	170.82	11.48	7.57	1.32
**21**	T1	46.72	0.60	0.87	0.51	0.81	0.43	5.26	242.93	15.26	9.33	1.32
**21**	T2	49.42	0.53	0.83	0.47	0.78	0.43	4.60	221.16	14.70	8.25	1.35
**21**	T3	50.95	0.57	0.85	0.45	0.74	0.41	4.48	223.00	14.72	8.75	1.42
**22**	T1	98.16	0.53	0.75	0.29	0.36	0.21	3.99	380.35	20.74	13.87	1.13
**22**	T2	91.83	0.59	0.77	0.31	0.39	0.22	4.06	348.90	19.09	13.69	1.16
**22**	T3	84.92	0.57	0.78	0.32	0.42	0.24	4.19	338.23	19.36	13.10	1.19
**23**	T1	60.89	0.40	0.59	0.43	0.58	0.37	4.66	275.31	14.99	10.07	1.11
**23**	T2	51.84	0.39	0.61	0.49	0.70	0.44	4.60	231.18	13.06	8.29	1.11
**23**	T3	55.17	0.41	0.69	0.45	0.70	0.42	4.11	215.15	12.78	7.48	1.13
**24**	T1	96.92	0.64	0.75	0.30	0.34	0.20	2.54	236.03	12.06	9.75	1.10
**24**	T2	108.21	0.70	0.74	0.28	0.29	0.17	2.35	242.52	11.85	10.65	1.09
**24**	T3	85.29	0.71	0.79	0.35	0.40	0.24	2.76	225.86	11.26	9.71	1.13

**25**	T1	48.89	0.49	0.80	0.48	0.78	0.44	4.34	209.18	13.42	7.27	1.22
**25**	T2	39.95	0.51	0.80	0.58	0.96	0.53	4.42	171.00	11.61	6.31	1.39
**25**	T3	39.43	0.51	0.84	0.57	0.98	0.53	4.29	165.17	11.78	6.11	1.37
**26**	T1	65.90	0.45	0.77	0.36	0.65	0.37	4.20	255.79	16.41	8.62	1.21
**26**	T2	55.69	0.49	0.79	0.44	0.77	0.43	4.32	225.19	14.72	8.07	1.24
**26**	T3	48.92	0.54	0.86	0.55	0.94	0.52	4.56	196.24	13.40	7.27	1.26
**27**	T1	70.20	0.74	0.91	0.42	0.62	0.33	4.52	286.81	16.21	11.82	1.15
**27**	T2	41.73	0.63	0.91	0.58	0.94	0.50	5.21	208.05	13.11	8.00	1.26
**27**	T3	38.32	0.69	0.98	0.63	1.02	0.52	6.04	223.99	14.43	8.74	1.25
**28**	T1	99.17	0.52	0.72	0.27	0.34	0.20	3.58	353.02	19.36	13.58	1.05
**28**	T2	105.33	0.49	0.69	0.26	0.32	0.19	3.31	342.24	18.89	13.02	1.05
**28**	T3	99.82	0.47	0.69	0.27	0.35	0.21	3.71	358.85	20.39	13.36	1.05
**29**	T1	30.75	0.64	0.98	0.74	1.56	0.78	7.50	212.83	15.04	8.09	1.47
**29**	T2	20.03	0.68	1.06	0.99	2.09	1.02	7.83	155.91	12.71	6.12	1.63
**29**	T3	18.99	0.64	1.03	1.02	2.17	1.08	7.58	143.05	11.86	5.42	1.57
**30**	T1	74.03	0.44	0.71	0.35	0.51	0.30	3.01	214.03	12.55	7.43	1.10
**30**	T2	61.83	0.61	0.86	0.41	0.60	0.33	3.09	183.46	10.86	7.05	1.21
**30**	T3	57.12	0.48	0.72	0.46	0.67	0.40	4.07	215.63	12.96	8.12	1.14
**31**	T1	92.18	0.54	0.67	0.32	0.37	0.23	2.84	241.13	12.39	9.48	1.04
**31**	T2	102.10	0.57	0.68	0.29	0.32	0.19	2.28	224.08	11.00	8.93	1.04
**31**	T3	91.26	0.61	0.71	0.33	0.36	0.21	2.50	208.00	10.50	8.67	1.06
**32**	T1	37.94	0.54	0.88	0.63	0.96	0.52	6.40	241.30	16.56	9.00	1.19
**32**	T2	34.52	0.56	0.87	0.69	1.05	0.57	6.53	224.18	15.03	8.55	1.19
**32**	T3	34.42	0.58	0.85	0.72	1.05	0.58	7.53	255.30	16.22	10.08	1.20
**33**	T1	34.48	0.82	1.11	0.67	1.17	0.56	7.83	271.60	17.25	11.52	1.33

**33**	T2	32.34	0.75	1.03	0.74	1.22	0.61	7.93	249.45	15.57	10.13	1.29
**33**	T3	26.86	0.60	0.84	0.89	1.36	0.7.5	8.06	216.13	12.62	8.12	1.22
**34**	T1	45.78	0.46	0.76	0.56	0.83	0.47	3.11	137.71	8.94	4.85	1.18
**34**	T2	41.80	0.49	0.76	0.63	0.90	0.52	3.48	137.50	8.62	5.07	1.16
**34**	T3	34.78	0.56	0.87	0.71	1.05	0.57	4.34	148.63	9.68	5.51	1.18
**35**	T1	88.84	0.67	0.76	0.33	0.37	0.21	3.08	268.41	13.53	11.22	1.17
**35**	T2	72.19	0.69	0.82	0.39	0.49	0.27	3.0	206.35	11.19	8.73	1.26
**35**	T3	63.70	0.70	0.83	0.44	0.55	0.30	3.32	206.92	11.42	8.86	1.23
**36**	T1	28.79	0.46	0.77	0.87	1.53	0.94	5.68	155.88	10.43	6.34	1.20
**36**	T2	18.92	0.43	0.76	1.14	2.12	1.23	6.13	115.35	8.13	4.36	1.21
**36**	T3	18.31	0.55	0.86	1.26	2.06	1.16	6.43	117.13	7.87	4.75	1.21
**37**	T1	29.93	1.01	1.02	1.00	1.51	0.75	10.32	258.91	15.66	12.38	1.72
**37**	T2	27.42	1.15	1.10	1.14	1.73	0.82	11.12	244.26	15.57	12.57	1.86
**37**	T3	21.47	1.25	1.10	1.22	1.80	0.86	12.03	241.51	15.35	13.66	2.12
**38**	T1	111.77	0.44	0.65	0.24	0.31	0.19	3.59	391.22	21.67	14.46	1.03
**38**	T2	135.29	0.48	0.64	0.22	0.25	0.15	3.27	400.36	21.30	15.80	1.04
**38**	T3	135.49	0.45	0.64	0.21	0.26	0.16	2.74	339.38	18.51	12.79	1.03
**39**	T1	35.68	0.70	1.02	0.78	1.17	0.60	6.35	203.10	13.32	8.56	1.26
**39**	T2	53.52	0.71	0.98	0.54	0.78	0.42	6.39	274.97	17.76	11.11	1.20
**39**	T3	56.21	0.66	0.88	0.51	0.77	0.42	5.74	280.85	17.51	12.87	1.24
**40**	T1	66.72	0.57	0.78	0.40	0.51	0.29	5.01	326.18	18.30	12.69	1.12
**40**	T2	73.65	0.60	0.80	0.37	0.47	0.26	3.99	284.23	16.39	11.53	1.14
**40**	T3	67.67	0.59	0.80	0.39	0.50	0.28	3.96	264.37	15.27	10.62	1.12
**41**	T1	30.38	0.43	0.90	0.66	1.41	0.75	5.53	162.83	14.31	5.76	1.38
**41**	T2	25.06	0.48	0.99	0.80	1.67	0.85	6.25	154.82	14.19	5.47	1.37

**41**	T3	22.39	0.60	1.12	0.92	1.90	0.90	6.53	142.45	13.51	5.61	1.47
**42**	T1	39.70	0.53	0.93	0.55	0.98	0.51	4.53	177.57	13.09	6.42	1.26
**42**	T2	32.94	0.66	1.06	0.67	1.16	0.57	5.51	180.15	13.62	7.14	1.33
**42**	T3	32.05	0.73	1.15	0.68	1.20	0.57	5.48	173.82	13.57	7.27	1.40
**43**	T1	47.45	0.71	0.99	0.61	0.97	0.49	5.11	222.66	15.80	10.01	1.37
**43**	T2	32.36	0.73	1.01	0.78	1.24	0.62	4.70	149.61	10.57	6.31	1.47
**43**	T3	33.19	0.75	0.98	0.82	1.19	0.60	4.90	156.73	11.29	7.08	1.52
**44**	T1	133.12	0.52	0.68	0.22	0.26	0.16	3.25	388.67	21.36	15.86	1.03
**44**	T2	135.65	0.49	0.66	0.21	0.25	0.15	2.89	370.23	20.38	14.83	1.02
**44**	T3	110.41	0.56	0.73	0.27	0.32	0.18	3.46	342.19	19.03	14.14	1.05
**45**	T1	53.68	0.40	0.70	0.44	0.70	0.41	5.27	277.79	17.69	9.36	1.15
**45**	T2	46.98	0.40	0.71	0.49	0.81	0.47	5.15	236.25	15.52	7.86	1.14
**45**	T3	41.94	0.42	0.74	0.56	0.91	0.53	5.42	222.56	14.73	7.50	1.16
**46**	T1	46.58	0.48	0.89	0.53	0.88	0.47	5.05	226.48	17.50	8.23	1.26
**46**	T2	33.52	0.50	0.94	0.67	1.15	0.60	5.26	175.19	15.57	6.73	1.46
**46**	T3	30.67	0.54	1.00	0.71	1.28	0.65	5.41	165.44	15.67	6.76	1.59
**47**	T1	58.67	0.52	0.64	0.51	0.62	0.38	7.28	397.91	20.85	16.51	1.12
**47**	T2	60.67	0.84	0.81	0.52	0.66	0.38	7.06	373.64	20.49	17.29	1.17
**47**	T3	46.97	0.55	0.74	0.53	0.83	0.49	5.25	235.61	15.04	9.05	1.18
**48**	T1	98.62	0.47	0.66	0.28	0.34	0.20	2.45	239.37	13.48	9.37	1.03
**48**	T2	92.88	0.46	0.67	0.29	0.37	0.22	2.61	232.37	13.33	8.91	1.05
**48**	T3	78.86	0.41	0.66	0.34	0.45	0.28	3.09	229.98	14.01	8.35	1.06
